# Plant Extracts for Sleep Disturbances: A Systematic Review

**DOI:** 10.1155/2020/3792390

**Published:** 2020-04-21

**Authors:** S. Guadagna, D. F. Barattini, S. Rosu, L. Ferini-Strambi

**Affiliations:** ^1^Opera Contract Research Organization Srl, A TIGERMED Company, Timisoara, Romania; ^2^University of Medicine and Pharmacy “Victor Babes”, Timisoara, Romania; ^3^IRCCS San Raffaele Scientific Institute, Department of Clinical Neurosciences, Neurology-Sleep Disorders Centre, Milan, Italy

## Abstract

**Background:**

Sleep complaints are common health issues in the general population. These conditions are associated with poorer physical and psychological activity, and they may have important social, economic, and personal consequences. In the last years, several food supplements with different plant extracts have been developed and are currently taken for improving sleep. *Study Objectives*. The aim of this study is to systematically review recent literature on oral plant extracts acting on sleep disorders distinguishing their action on the different symptoms of sleep complaints: difficulty in initiating or maintaining sleep, waking up too early, and quality of sleep.

**Methods:**

We searched the PubMed database up to 05/03/2020 based on data from randomized, double-blind, placebo-controlled trials, noncontrolled trials, and cohort studies conducted in children and adult subjects. The search words used contained the following terms: oral food supplement and sleep disorders and the like. The most studied compounds were further analyzed with a second search using the following terms: name of the compound and sleep disorders. We selected 7 emerging compounds and 38 relevant reports.

**Results:**

Although nutraceutical natural products have been used for sleep empirically, there is a scarcity of evidence on the efficacy of each product in clinical studies. Valerian and lavender were the most frequently studied plant extracts, and their use has been associated (with conflicting results) with anxiolytic effects and improvements in quality and duration of sleep.

**Conclusions:**

Sleep aids based on plant extracts are generally safe and well tolerated by the population. More high-quality research is needed to confirm the effectiveness of food supplements containing plant extracts in sleep complaints; in particular, it would be interesting to evaluate the association between plant extracts and sleep hygiene guidelines and to identify the optimal products to be used in a specific symptom of sleep complaint, giving more appropriate tools to the medical doctor.

## 1. Introduction

Insomnia is defined as dissatisfaction with sleep quality or quantity in addition to at least one other symptom among difficulty initiating sleep, difficulty maintaining sleep, or early morning awakening with inability to return to sleep [1]. Occasional insomnia is a very common disturb that has been reported to be experienced by about 30% of the U.S. general population [1–3]. Sleep disorders have an important societal and economic impact, with a consequent reduction in labour productivity or increased risk of accidents [4–6]. Chronic insomnia is also a risk factor for a variety of significant health problems, such as cardiovascular disease [7, 8], diabetes [9], and obesity [10], as well as bad mood and cognitive dysfunction [11–13]. Almost half of the individuals with sleep problems had never taken any steps to resolve them, and the majority of respondents had not spoken with a physician about their problems. Of those individuals who had consulted a physician, drug prescriptions had been given to approximately 50% in Western Europe and the USA [14]. The commonly used sleep aids based on benzodiazepine and non-benzodiazepine hypnotic drugs are often related to negative side effects such as daytime drowsiness, dependency, depression, hypnotic-withdrawal insomnia, and even excess mortality [15]. Moreover, there are limited data on long-term efficacy of hypnotic drugs [16]. Given these concerns and an increasing patient preference for nonpharmacological treatments [17], it is important to offer patients with insomnia evidence-based nonpharmacologic alternatives that may improve their sleep.

As defined in the Dietary Supplement Health and Education Act of 1994 (DSHEA), a dietary supplement is “a product (other than tobacco) intended to supplement the diet that bears or contains one or more dietary ingredients, including a vitamin, a mineral, an herb or other botanical, an amino acid, a dietary substance for use by humans to supplement the diet by increasing the total dietary intake of any of the aforementioned ingredients [18].” A growing body of evidence has shown promising results for these compounds in supporting health and body functions [19]. In particular, several dietary supplements are popularly used for sleep disorders [20], also in addition to other remedies (e.g., sleep hygiene and mind-body therapies) [21]. Moreover, no golden standard therapy is recommended to treat mild sleep disorders related to specific sleep stages (starting, maintaining, and ending sleep) [22, 23].

Our aim in this study was to systematically review recent literature on plant extracts and nutraceuticals administered orally and acting on sleep-related disorders. In particular, we differentiated the interventions and the outcomes of the studies based on the different sleep disorders (difficulty in initiating or maintaining sleep, quality and quantity of sleep, and waking up too early) and reviewed the available clinical data of the 7 most studied natural products: valerian, lavender, chamomile, hop, St. John's wort, hawthorn, and rosemary.

## 2. Materials and Methods

**Figure 1:**
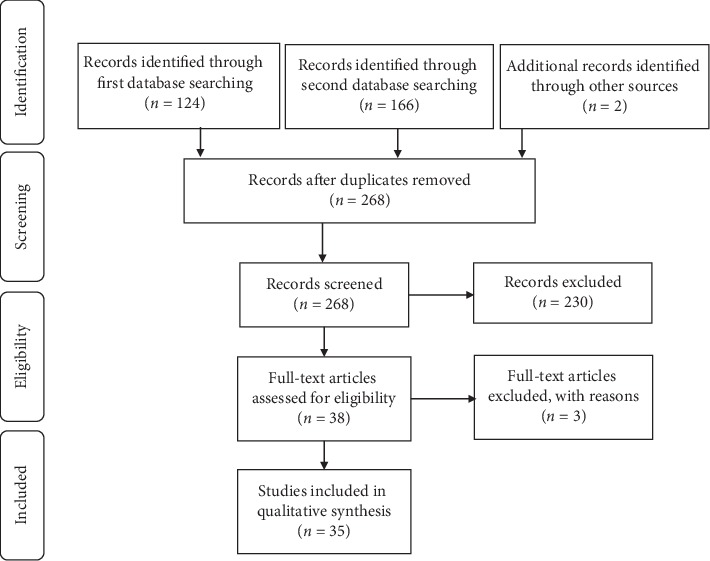
Flow diagram of information according to PRISMA 2009 [73].

A literature search was performed using a primary medical search engine the PubMed database considering all articles published up to 05/03/2020; the registered review protocol can be found at: https://www.crd.york.ac.uk/PROSPEROFILES/126991_PROTOCOL_20190301.pdf. The review was registered on PROSPERO (international prospective register of systematic reviews in https://www.crd.york.ac.uk/prospero/), registration number CRD42019126991. The inclusion criteria were randomized, double-blind, placebo-controlled trials, noncontrolled trials, and cohort studies. We used the following search terms to search the PubMed register: (Oral food supplement) OR (Oral nutraceutical) OR (Oral natural products) AND (Sleep disorders) OR (Insomnia) AND “humans” [Filter] AND “English”[Filter]. The most studied compounds were singled out and further analyzed with a second search using the terms: (name of the compound) AND (Sleep disorders) OR (Insomnia) AND “humans”[Filter] AND “English”[Filter]. Only articles written in English and only studies conducted on humans were selected for this review. Additionally, the same research criteria were applied also for the Spanish language but no additional references were found. We contacted the study authors to retrieve the full article where only the abstract was available. We selected 7 emerging compounds and 35 relevant reports, excluding duplicates, nonrelevant articles, reviews, and works with no full article available (Figure 1). Information was extracted from each included trial in view of: (1) type of food supplement for sleep disorders (herbal component, dose, length of the treatment, and additional substances) and (2) clinical endpoints considering the different stages of sleep and sleep problems: sleep latency, sleep maintenance, quality of sleep, and quantity of sleep. Finally, the risk of bias of individual studies was considered both at study or outcome level, and the Jadad scale [24] for quality rating was used to assess the quality of works. Parameters considered were randomization, blinding, withdrawals, sample size, quality of data reported, and statistical analysis. Publication bias and selective reporting within studies are likely to be affecting the selected literature for this review.

## 3. Results

### 3.1. Valerian (*Valeriana officinalis*)

Valerian is the most studied plant for sleep disorders. We selected 17 articles on this subject to be included in the present review. The results of clinical trials performed to test valerian as a sleep aid are controversial and conflicting. Several studies showed an improvement in sleep quality [25–32] after administration of valerian at doses ranging from 160 to 600 mg a day. Differently, other studies reported no improvement in sleep quality (measured with Pittsburgh sleep quality index, PSQI, or perceived) [33–35]. Additionally, valerian was shown to reduce wake time after sleep onset [25, 27], to improve sleep latency and duration [36, 37], and to ameliorate insomnia severity score [38]. Conversely, a study from Jacobs and collaborators showed no changes in the insomnia severity score (ISI) compared to placebo [39]. Diaper and collaborators in a small study observed no changes in polysomnographic parameters or psychometric measures after one dose of 300 mg or 600 mg of valerian [40], and Coxeter reported no changes in total sleep time or number of nocturnal awakenings in the participants' responses in a *n*-of-1 analysis of 24 subjects [41].

Some trials investigated the possible mechanism of action of the effect of valerian as sleep aid. The study from Mineo and collaborators showed that a single oral dose of *Valeriana officinalis* extract caused a significant reduction in intracortical facilitation, a change associated with reduced anxiety [42].

### 3.2. Lavender (*Lavandula*)

In 2010, Woelk and collaborators showed in a double-blind, randomised study with 77 subjects that silexan, an oral lavender oil capsule preparation, is as effective as lorazepam in adults with generalised anxiety disorder (GAD). Hamilton Anxiety Rating Scale (HAM-A) scores for anxiety and sleep diary scores demonstrated comparable positive effects [43]. Two studies from Kasper et al. in 2010 [44] and 2015 [45] with a dose of 80 mg of silexan showed significant improvement in sleep quality (PSQI) and anxiety (HAM-A) compared to placebo. Finally, an open-label trial with silexan and 47 participants indicated a reduction of nocturnal awakening frequency and duration after 6 weeks of assumption of the food supplement [46].

### 3.3. Hop

A double-blind, randomized placebo-controlled trial on 171 volunteers with sleep difficulties reported no significant changes in sleep quality (PSQI) after assumption of the LZComplex3 (hops 500 mg) for 2 weeks [47]. Another study with 101 volunteers with chronic primary insomnia assuming two gelatine capsules of Cyclamax® (50 mg hop) per day for a month, showed no effects on sleep quality Leeds sleep evaluation questionnaire (LSEQ), melatonin metabolism, and sleep-wake cycle [48].

### 3.4. Chamomile

A study on sixty elderly people who assumed chamomile extract capsules (200 mg) twice a day for 28 consecutive days reported improvements in general sleep quality and sleep latency (PSQI) [49].

Chang and colleagues conducted a study on the effects of drinking chamomile tea on sleep quality in sleep disturbed postnatal women and found a modest improvement in the PSQS (postpartum sleep quality scale) subscale “physical symptoms-related sleep inefficiency” at 2 weeks but not at 4 weeks [50]. Finally, Zick and colleagues performed a pilot trial with 34 subjects with DSM-IV primary insomnia and found no significant improvements in ISI and PSQI [51].

### 3.5. Hawthorn (*Crataegus oxyacantha*)

A double-blind, randomized, placebo-controlled study with 264 subjects showed a reduction in total and somatic Hamilton scale scores for anxiety (*p*=0.005) [52].

No trial investigated directly the effects of hawthorn in sleep disorders.

### 3.6. St. John's Wort (*Hypericum perforatum*)

Many clinical trials tested the herb St. John's wort for mild to moderate depression. Al-Akoum et al. reported that 900 mg of St. John's wort decreased scores of the sleep problem scale compared with placebo in perimenopausal women after 12 weeks of oral administration [53].

No trial investigated directly the effects of St. John's wort in sleep disorders.

### 3.7. Rosemary (*Rosmarinus officinalis* L.)

A randomized clinical trial from Nematolahi and collaborators on subjects who received 500 mg of rosemary showed a significant improvement in sleep quality using the PSQI after one month, but not on sleep latency and sleep duration [54].

### 3.8. Valerian and Hops

Some clinical trials investigated the combined effect of different plant extracts on sleep related problems; the most studied combination of ingredients is valerian and hop.

Dimpfel and Suter reported that a single dose administration of a valerian and hop fluid extract improved total sleep time and quality of sleep in poor sleepers [55]. Maroo et al. tested a mixture of valerian, passion flower, and hop extract and found significant improvements in sleep time, sleep latency, number of nightly awakenings, and insomnia severity index after a 2 week treatment [56]. Koetter et al. showed a reduction of sleep latency after a treatment period which lasted for 4 weeks with a fixed extract combination of valerian and hop [57].

Conversely, Morin et al. found very modest effects of a valerian and hop combination and only in quality of life scores [58]. Finally, a study from Sun investigated the effects of a mixture of herbal extracts (kava, hop, valerian, and many others) on sleep disturbance in menopausal women. The authors reported that the formula significantly reduced global PSQI score and scores in five components (sleep quality, sleep latency, sleep duration, sleep disturbance, and daytime dysfunction) [59].

Table 1 shows the 35 studies included in this review.

Table 2 summarizes the effects of the different compounds on sleep parameters.

## 4. Discussion

Sleep disturbances are widespread and affect a high percentage of the general population [1–3].

Food supplements use for sleep complaints is extensively adopted. In a survey in the province of Quebec on almost 1000 subjects, 18.5% participants reported having used natural products as sleep aids [60].

The most commonly used plant extracts for insomnia are valerian, chamomile, and lavender. In general, the selected studies showed a good quality with an average of 3, 4 points in the Jadad scale (0–5) [24] for quality rating, only 6 studies were evaluated with a score <3 and 10 studies with a score of 5. Many studies, however, are limited by small numbers of participants and, in some instances, inadequate design and sparse use of objective measurements. As mentioned by Fernández-San-Martín et al. in a metanalysis on lavender use for sleep disturbances [61], a wide range of dosages and types of preparations are often used and most measurement methods are open for interpretation. When the analysis is performed with quantifiable variables (latency time in minutes and sleep quality measured with VAS), no significant improvement is frequently found.

There is preliminary but conflicting evidence suggesting valerian and lavender as possible sleep aids for mild problems of quality of sleep, sleep latency, total sleep time, and waking up after sleep onset. Notably, the studies contrasted the efficacy of valerian rated with a high Jadad score (5 studies with score of 5, Table 1). On the other hand, a recent meta-analysis of randomized, placebo-controlled trials showed a significant effect of lavender oil (Silexan) in reducing the HAMA total score for psychic and somatic anxiety and for observer-assessed and self-assessed anxiety [62].

Valerian activity on sleep disturbances has been attributed to the presence of isovaleric acids and valepotriates with reported calming action [63] and GABA reuptake inhibition with sedative effects [64]. Considering the data presented in the literature, valerian seems more effective for chronic insomnia than acute episodes.

The main components of the lavender preparations are linalyl acetate and linalool [65]. In mice, these components led to anticonvulsant effects [66], depression of motor activity, and calming effects [65].

Sparse or no scientific data were found to support the efficacy of most products as hypnotics, including chamomile, hop (alone), hawthorn, St. John's wort, and rosemary. Notably, one recently published systematic review and a meta-analysis indicated chamomile as efficacious and safe for improving sleep quality and generalized anxiety disorders but highlighted scarce effect for insomnia [67, 68].

Other plant extracts have been proposed and tested in clinical trials. Kava kava has been well studied and has showed good results in reducing anxiety and hypnotic effects [69], but because of its hepatotoxic effects, the prescription has been forbidden [64]. In addition, extracts from poppy, passionflower, and lemon balm (Melissa) to mention the most popular ones have been investigated in sleep disturbances, but so far, the amount of data is not sufficient to evaluate their effect on these disorders.

Unfortunately, not many trials tested the efficacy of a combined nonpharmacological intervention based on the administration of plant extracts and standardized sleep hygiene in subjects with mild to moderate insomnia. This combination could improve the efficacy in many trials where a single herbal extract was tested. In support of this hypothesis, a study by Maroo et al. [56] showed that a composition of valerian, passionflower, and hop improved total sleep time, sleep latency, number of nightly awakenings, and insomnia severity index. Moreover, a pilot study testing a combination of melatonin, vitamin B6, and various plant extract showed a positive result in sleep quality, sleep onset latency, and total sleep duration [70].

The management of sleep complaints relies on both pharmacological and nonpharmacological approaches. The last years evidenced a decrease in using sedative and hypnotic drugs to treat these conditions. On the other hand, the population and the medical community are considering food supplements and other nonpharmacological approaches in the management of mild and recent insomnia [71]. To date, however, as pointed out in various recent systematic reviews [21, 72], more high-quality research is needed to confirm the effectiveness of plant extracts in sleep disorders, in particular for chronic conditions and in association with complementary and alternative medicine, such as sleep hygiene and mind-body therapies.

## Figures and Tables

**Table 1:** List of studies with selected compounds.

Compound	First authors (year published)	Design	Total patients	Intervention	Reported outcomes/results	Journal	Jadad scale
Valerian	Taavoni 2013 [30]	Triple-blind, Randomized placebo-controlled trial	100	160 mg of essence of valerian and lemon balm	Improvement in sleep quality (PSQI)	Complementary Therapies in Clinical Practice	3
	Taavoni 2011 [29]	Triple-blind, Randomized placebo-controlled trial	100	530 mg valerian extract	Improvement in sleep quality (PSQI)	Menopause	3
	Barton 2011 [35]	Double-blind Randomized placebo-controlled trial	119	450 mg of valerian	No improvement in sleep quality (PSQI)	The Journal of Supportive Oncology	3
	Cuellar 2009 [34]	Triple-blind, Randomized placebo-controlled trial	37	800 mg of valerian	No improvement in sleep quality (PSQI)	Alternative Therapies in Health and Medicine	5
	Waldschütz 2008 [37]	Open-label, prospective cohort study	409	Doses were at physicians' judgments	Improved sleep latency and duration	The Scientific World Journal	1
	Oxman 2007 [33]	Web-based randomized placebo-controlled trial	405	200 mg extract per tablet (valerina forte).	No improvement in sleep quality perceived	PLoS One	5
	Müller 2006 [31]	Open, multicentre study	918	Euvegals forte (160 mg valerian root)	Reduced dyssomnia	Phytomedicine	1
	Jacobs 2005 [39]	Internet-based randomized, placebo-controlled trial	391	2 valerian softgel capsules (3.2 mg of valerenic acids)	No changes in ISI	Medicine	5
	Diaper 2004 [40]	Placebo-controlled three way crossover	16	Acute valerian 300 mg or valerian 600 mg	No changes in EEG parameters or psychometric measures	Phytotherapy Research	5
	Coxeter 2003 [41]	Randomized n-of-1 trials	24	225 mg V. officinalis root and rhizome extract	No changes in total sleep time or number of night awakenings	Complementary Therapies in Medicine	5
	Ziegler 2002 [28]	Randomized, double-blind, comparative trial	202	600 mg/die valerian extract	Improvement in sleep quality	European Journal of Medical Research	NA
	Poyares 2002 [27]	Double-blind, Randomized placebo-controlled trial	37	100 mg valerian (valmane)	Improvement in sleep quality and wake time after sleep onset	Progress in Neuropsychopharmacology and Biological Psychiatry	3
	Herrera-Arellano 2001 [26]	Double-blind, cross-over, placebo-controlled study	20	450 mg of valerian	Improvement in sleep quality and morning sleepiness	Planta Medica	3
	Wheatley 2001 [38]	Cross-over study compared to kava	19	600 mg of valerian	Improvement in insomnia severity scores	Phytotherapy Research	1
	Donath 2000 [36]	Double-blind, cross-over, placebo-controlled study	16	300 mg dry extract valerian (sedonium)	Improvement in slow-wave sleep latency	Pharmacopsychiatry	4
	Lindahl 1988 [32]	Double-blind, Randomized placebo-controlled trial	27	400 mg of valepotriates	Improvement in sleep quality	Pharmacology Biochemistry and Behavior	4
	Leathwood 1982 [25]	Double-blind, Randomized placebo-controlled trial	128	400 mg valerian for 3 days	Improvement in sleep quality and wake time after sleep onset	Pharmacology Biochemistry and Behavior	4
Lavender	Kasper 2015 [45]	Double-blind, Randomized placebo-controlled trial	170	80 mg of silexan daily for 10 weeks	Improvement in sleep quality (PSQI) and anxiety (HAMA)	European Neuropsychopharmacology	4
	Uehleke 2012 [46]	Open-label, exploratory trial	47	1 × 80 mg/day silexan over 6 weeks	Reduced waking-up frequency and duration	Phytomedicine	1
	Kasper 2010 [44]	Double-blind, Randomized placebo-controlled trial	221	80 mg of silexan daily for 10 weeks	Improvement in sleep quality (PSQI) and anxiety (HAMA)	International Clinical Psychopharmacology	5
	Woelk 2010 [43]	Double-blind, Randomized lorazepam-controlled trial	77	1 × 80 mg/day silexan over 6 weeks	Improvement in anxiety (HAMA) and sleep quality (sleep diary)	Phytomedicine	4
Hop	Scholey 2017 [47]	Double-blind, Randomized placebo-controlled trial	171	LZComplex3 (lactium, Zizyphus, Humulus lupulus, magnesium, and vitamin B6) hop 500 mg for 2 weeks	No changes in sleep quality (PSQI)	Nutrients	5
	Cornu 2010 [48]	Double-blind, Randomized placebo-controlled trial	101	Two gelatine capsules of Cyclamax® (50 mg hop, 260 mg soya oil, 173 mg *Cannabis sativa*) per day for a month	No effects on sleep quality (LSEQ), melatonin metabolism, and sleep-wake cycle	BMC Complementary and Alternative Medicine	3

Chamomile	Adib-Hajbaghery 2017 [49]	Single-blind randomized controlled trial	60	200 mg twice a day for 28 days	Improvement in general sleep quality and sleep latency (PSQI)	Complementary Therapies in Medicine	3
	Chang 2016 [50]	A single-blinded, randomized controlled	80	one cup of chamomile tea per day for 2 weeks	Improvement in PSQS	Journal of Advanced Nursing	3
	Zick 2011 [51]	Double-blind, randomized, placebo-controlled pilot trial	34	270 mg of chamomile twice daily for 28 days	No significant improvement in ISI and PSQI	BMC Complementary and Alternative Medicine	5

Hawthorn	Hanus 2003 [52]	Double-blind, randomized, placebo-controlled trial	264	150 mg twice daily for 3 months	Reduction in Hamilton Anxiety Scale	Current Medical Research and Opinion	4

St. John's wort	Al-Akoum 2009 [53]	Pilot double-blind, randomized	47	900 mg three times daily	Improvement in general sleep quality (SPS)	Menopause	5

Rosemary	Nematolahi 2018 [54]	Double-blinded randomized placebo-controlled trial	68	500 mg rosemary	Improvement in sleep quality (PSQI)	Complementary Therapies in Clinical Practice	3
Valerian + hop	Maroo 2013 [56]	Double-blinded randomized Zolpidem-controlled trial	78	300 mg valerian, 80 mg passion flower, and 30 mg hop	Improvement in total sleep time, sleep latency, number of nightly awakenings, and ISI	Indian Journal of Pharmacology	5
	Dimpfel 2008 [55]	Double-blinded randomized placebo-controlled trial	42	460 mg of valerian and 460 mg of hop single dose	Improvement in sleep quality (deep sleep) and sleep quantity	European Journal of Medical Research	3
	Koetter 2007 [57]	Double-blinded randomized placebo-controlled trial	27	500 mg of valerian and 120 mg of hop for 4 weeks	Improvement in sleep latency	Phytotherapy Research	2
	Morin 2005 [58]	Double-blinded randomized placebo-controlled trial	184	2 tablets of 187 mg of valerian and 41.9 mg of hop for 28 days	Improvement in quality of life (physical component)	Sleep	3
	Sun 2003 [59]	Open, noncomparative trial	72	200 mg valerian, 100 mg hop, kava and other components	Improvement in sleep quality, sleep latency, sleep duration, sleep disturbance (PSQI)	The Journal of Alternative and Complementary Medicine	0

SPS = sleep problem scale, PSQI = Pittsburg sleep quality inventory, PSQS = postpartum sleep quality scale, LSEQ = Leeds sleep evaluation questionnaire, and ISI = insomnia severity index.

**Table 2:** Sleep outcomes.

Compounds	SL	WASO	TST	QOS
Valerian	✓	✓	✓	✓
Lavender	✓	✓	✓	✓
Hop				
Chamomile	✓			✓
Hawthorn				
St. John's wort				✓
Rosemary				✓

SL = sleep latency, WASO = wake after sleep onset, TST = total sleep time, and QOS = quality of sleep.

## Supplementary Materials

Supplementary Materials PRISMA 2009 Checklist.
